# Supplementary material for: Comprehensive profiling of neutralizing polyclonal sera targeting coxsackievirus B3
Source: Nat Commun. 2023 Oct 12;14:6417. doi: 10.1038/s41467-023-42144-2 (PMC10570382; doi:10.1038/s41467-023-42144-2)
Supplement: Supplementary file 1 — Supplementary Information [file 41467_2023_42144_MOESM1_ESM.pdf]

Supplementary Information

Figures S1-S7 and Tables S1-S6.

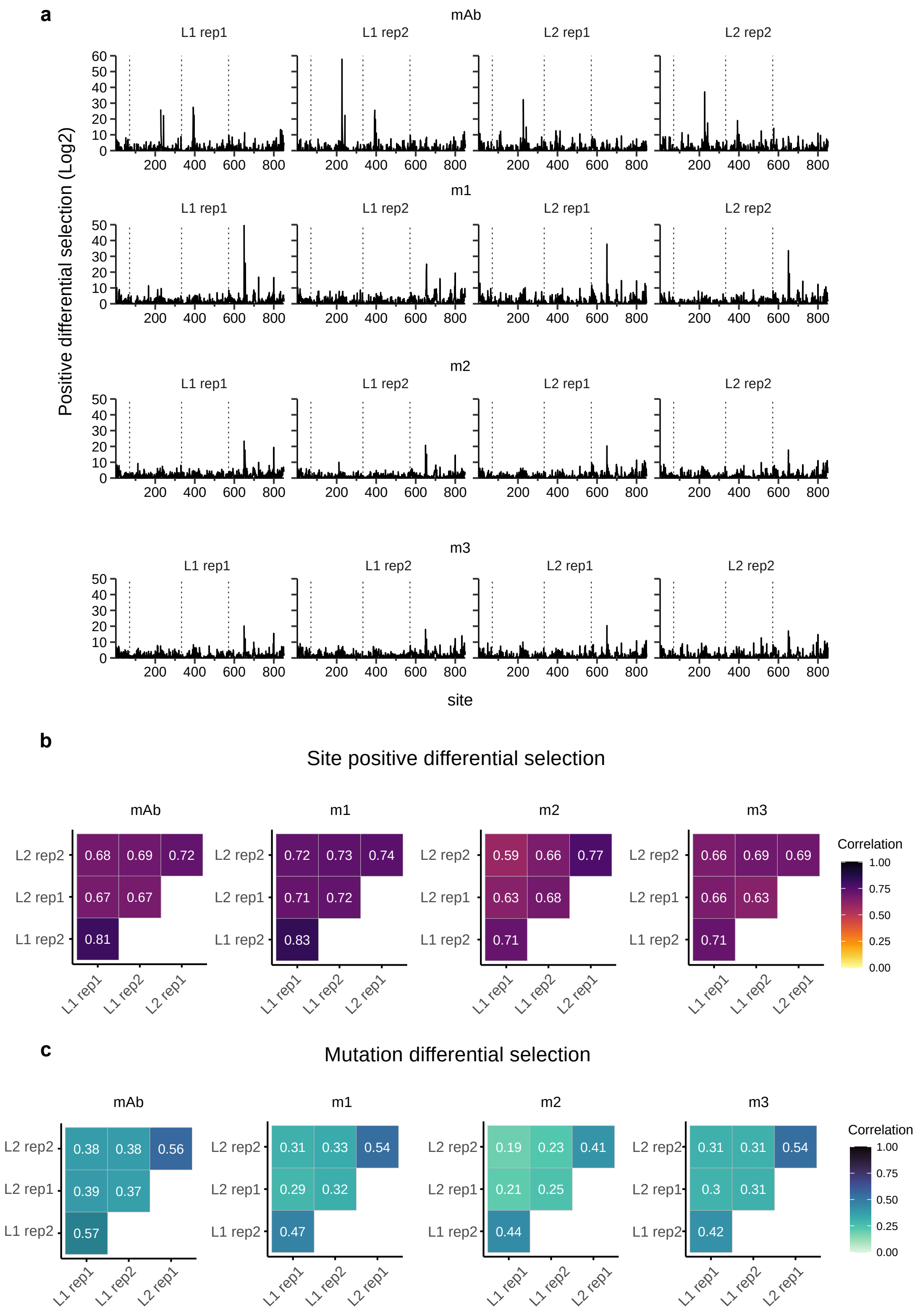

Figure S1. Related to Figures 1 and 2. Technical and biological replicates of the mutational antigenic profiling of a mAb and mouse sera. (a) Positive differential selection profiles of antigenic

profiling of the mAb and three mouse sera samples (m1, m2, and m3). Two replicates (rep1 and rep2) were performed for two independent virus libraries: library 1 and library 2 (L1 and L2, respectively). **(b,c)** Matrices of Pearson correlation coefficients for site positive differential selection **(b)** and mutation differential selection **(c)** between biological and technical replicates.

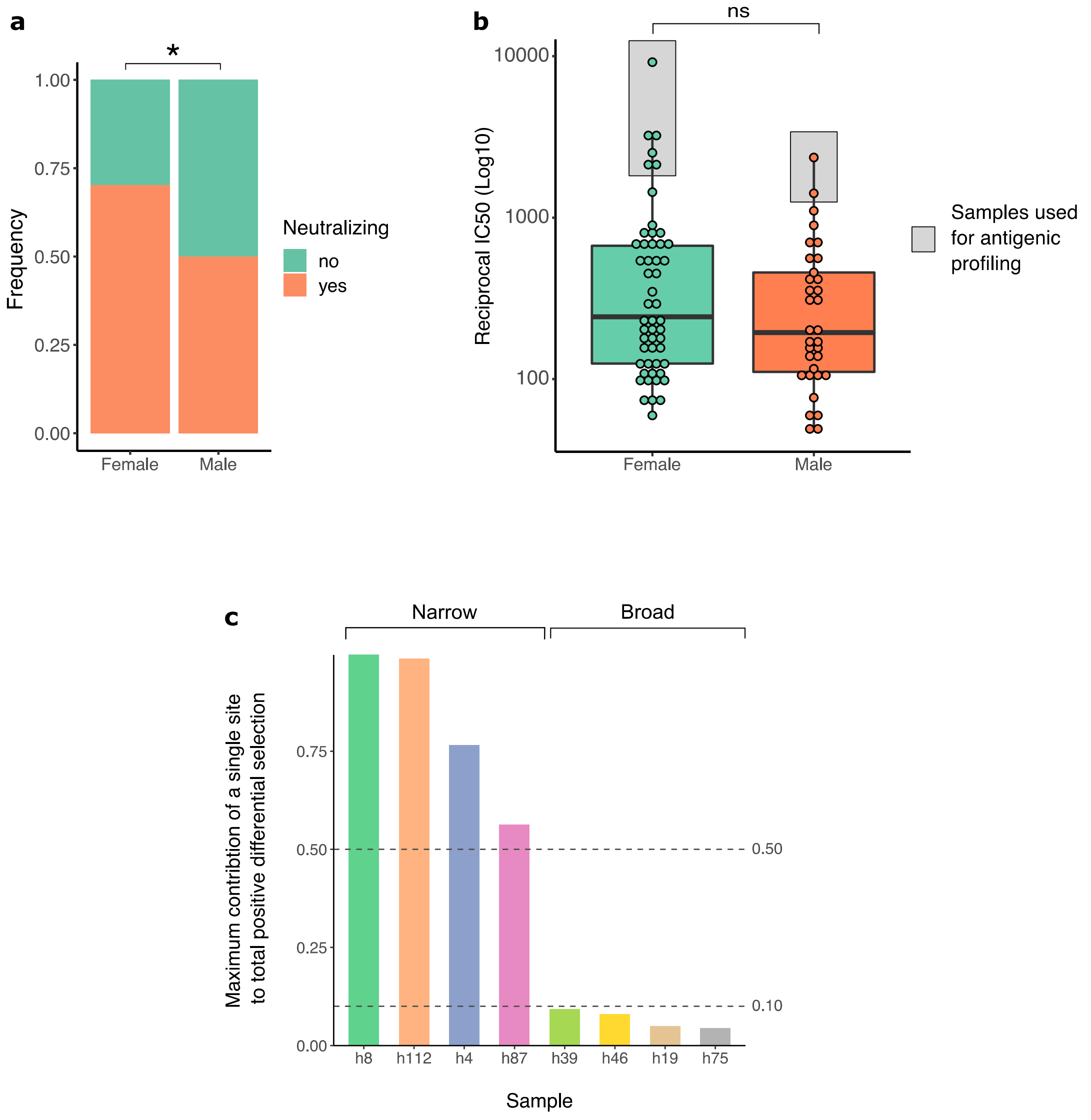

Figure S2. Related to Figures 3 and 4. Screening for CVB3 neutralizing sera in healthy donors and classification of antigenic profiles. (a) Frequency of neutralizing sera, defined as having a reciprocal IC50 > 40, by sex. The prevalence of neutralizing samples was 60% (85/140). Female donors had higher seroprevalence (neutralizing samples female=52/74, male=33/66; p-value = 0.01605 by Fisher's exact test). **(b)** Reciprocal IC50 values of neutralizing samples per sex (ns or p > 0.05 by Wilcoxon's test). Samples used for the antigenic profiling experiments are highlighted with gray rectangles (top 6 female and top 2 male neutralizing sera). **(c)** Classification of the antigenic profiles of human sera as narrow or broad according to the maximum contribution of any single site to escape. Narrow or broad profile sera are defined as those where the maximum contribution of any site to escape comprises >50% or <10% of all positive differential selection observed for that sample, respectively.

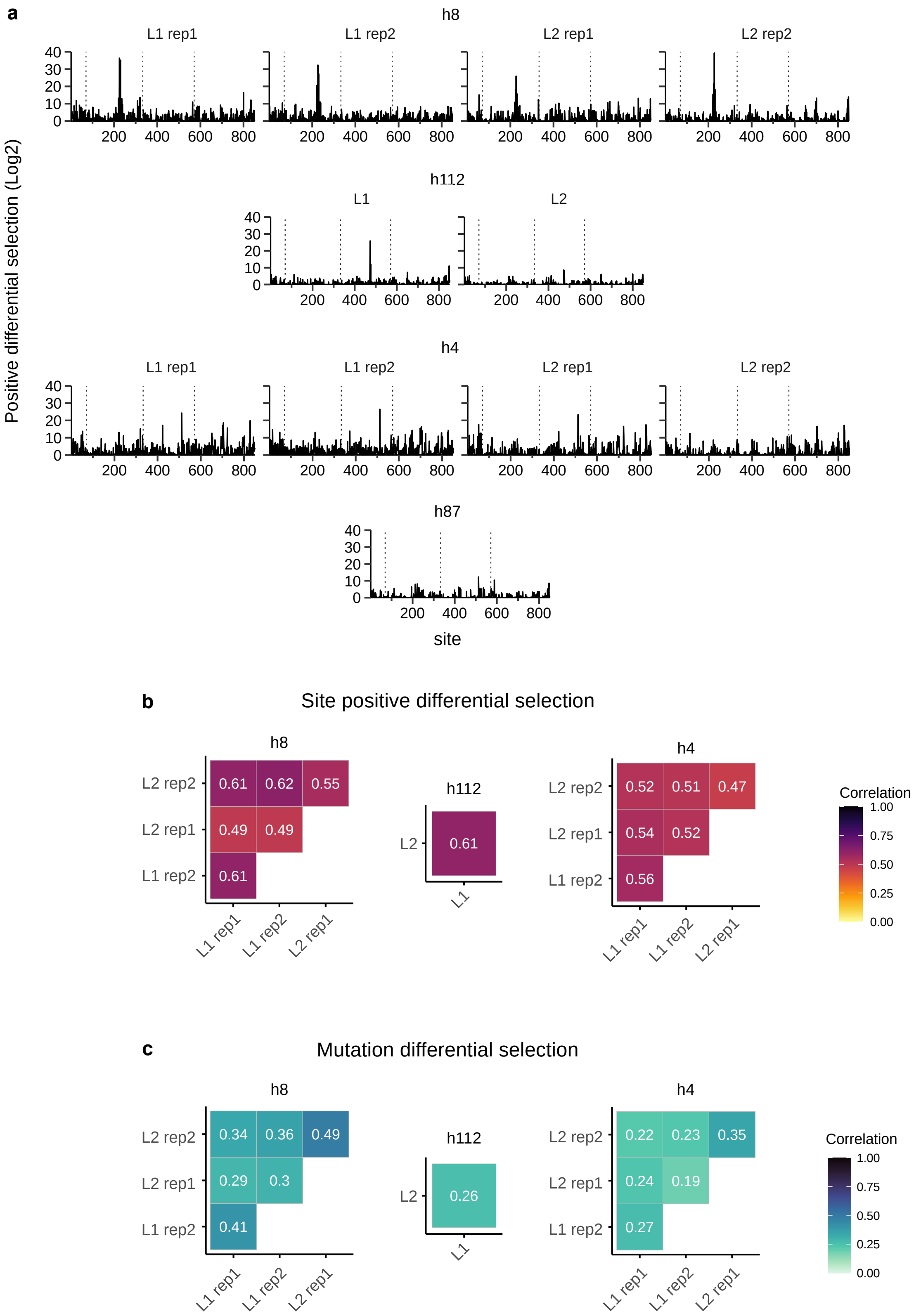

Figure S3. Related to Figure 3. Technical and biological replicates of the mutational antigenic profiling of narrow human sera samples. (a) Positive differential selection profiles of the narrow human sera samples (h8, h112, h4, h87). One (h112) or two (h4, h8) replicates (rep1 and rep2) were performed for two independent virus libraries: library 1 and library 2 (L1 and L2, respectively). Sample h87 was only analyzed with L2. **(b,c)** Matrix of Pearson correlation coefficients of site positive differential selection **(b)** and mutation differential selection **(c)** between biological and technical replicates.

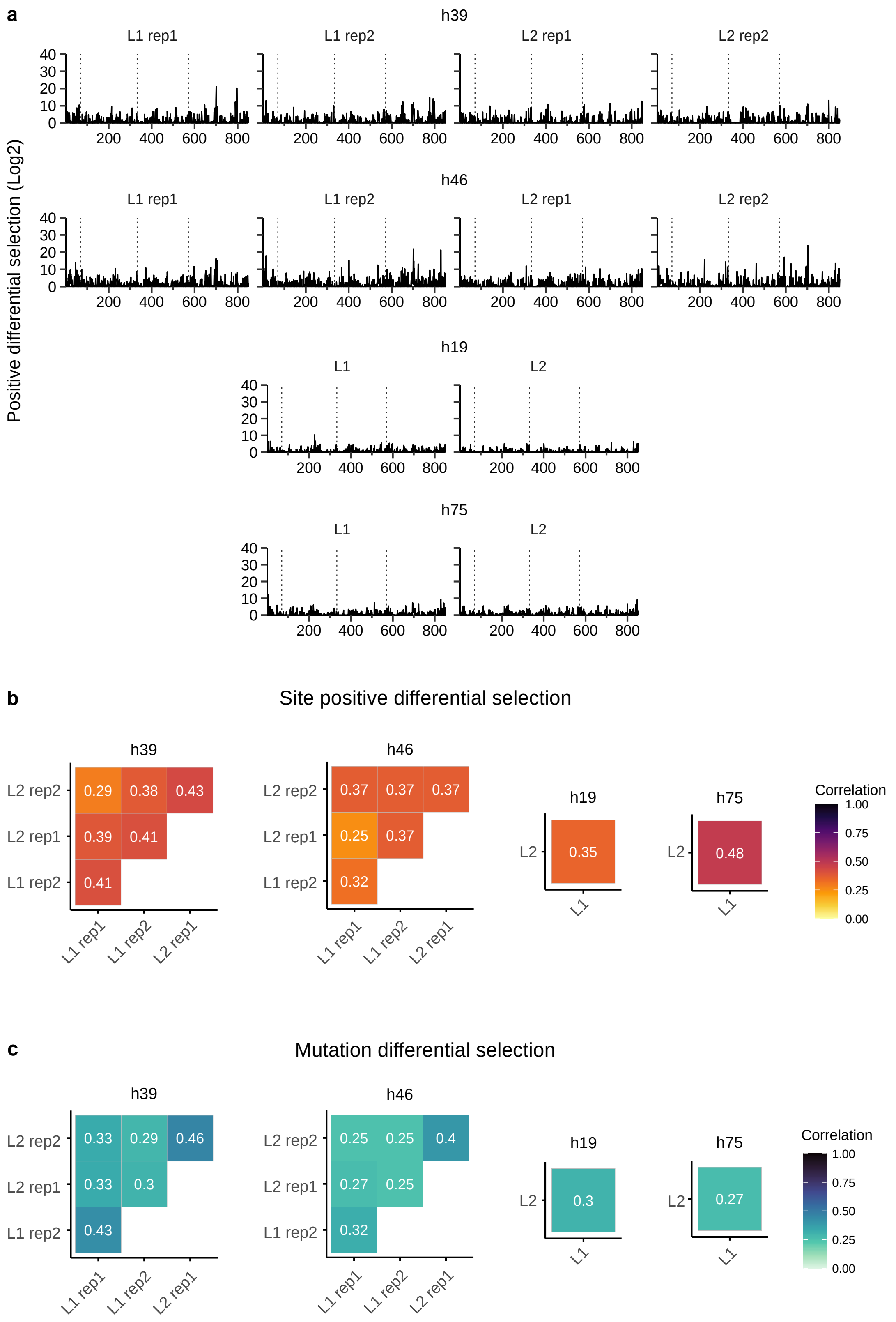

Figure S4. Related to Figure 4. Technical and biological replicates of the mutational antigenic profiling of broad human sera. (a) Positive differential selection profiles of broad human sera (h39, h46, h19, h75). One (h19, h75) or two (h39, h46) replicates (rep1 and rep2) were performed for two independent virus libraries: library 1 and library 2 (L1 and L2, respectively). **(b,c)** Matrix of Pearson correlation coefficients of site positive differential selection **(b)** and mutation differential selection **(c)** between biological and technical replicates.

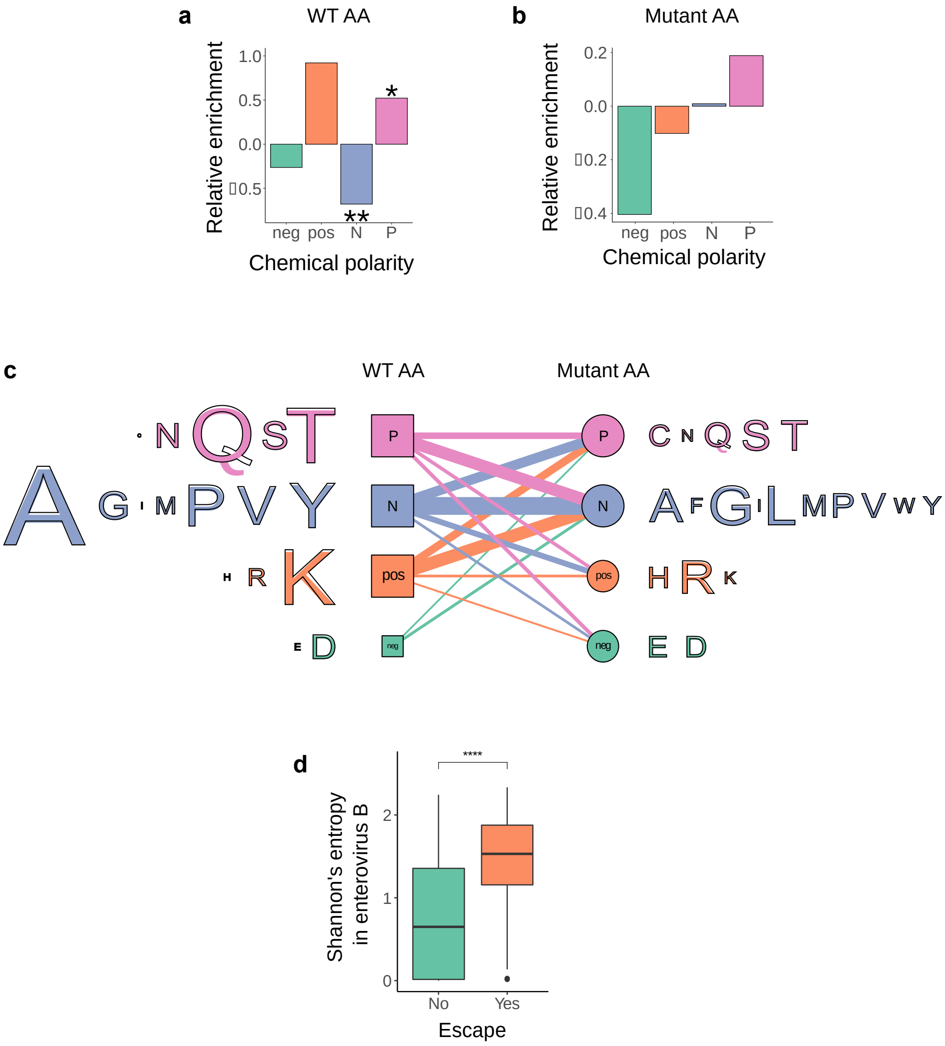

Figure S5. Related to Figure 5. Analysis of mutations that confer escape from human polyclonal sera. (a) The relative enrichment of the chemical polarity of amino acids in sites conferring escape versus surface exposed residues where no escape is observed. **(b)** The relative enrichment of the chemical polarity of individual amino acids conferring escape versus mutations in the same residues which do not confer escape. **(c)** Bipartite network analysis of WT and mutant AAs where escape is observed. Edge width is proportional to the number of occurrences. Vertex size is proportional to the number of connected edges. The size of amino acid letters is proportional to the number of occurrences. Color code according to physicochemical properties is the same as that used in Figure 5. **(d)** Shannon's entropy of surface exposed residues conferring escape from neutralization and surface exposed sites where no escape is observed. Shannon's entropy was obtained as described in Mattenberger et al., eLife, 2021. P=polar, N=non-polar, pos=positively charged, neg=negatively charged.

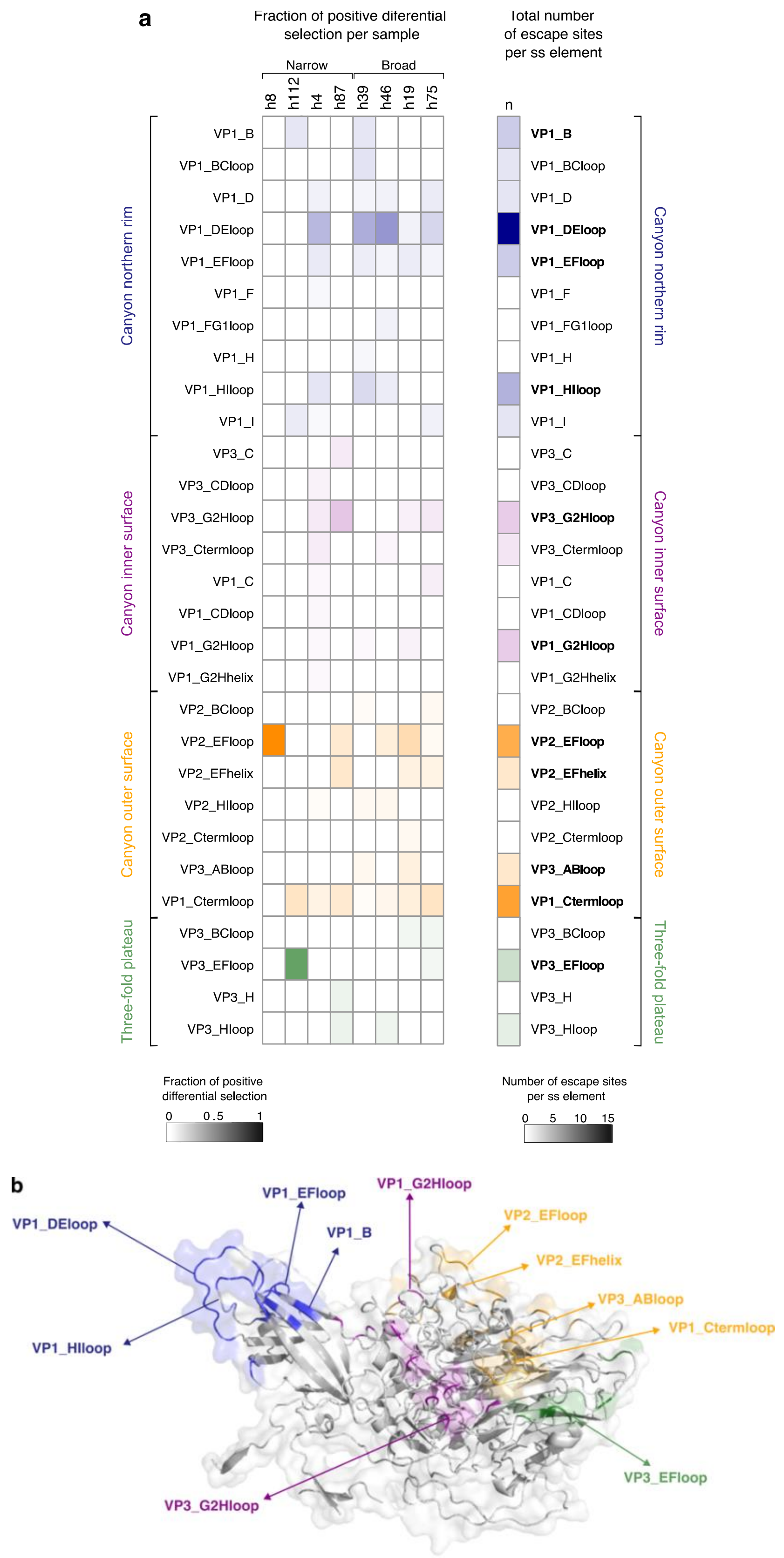

Figure S6. Related to Figure 6. Structural distribution of top human escape sites. (a) Heatmap representation of the total (right) or per sample (left) positive differential selection by secondary structure element and capsid region. Sites of escape were defined as surface exposed residues with a positive differential selection value above the mean+2SD value per sample (n=76, https://github.com/RGellerLab/CVB3-Antigenic-Profiling/tree/main/10_analysis_sites_muts). Secondary structure elements with a higher number of escape sites (n ≥ 3) are highlighted in bold. **(b)** Structural

representation of the most prevalent secondary structure elements. The capsid monomer of the CVB3 strain Nancy is represented with the most prevalent secondary structure elements colored according to their structural location colored as in (a).

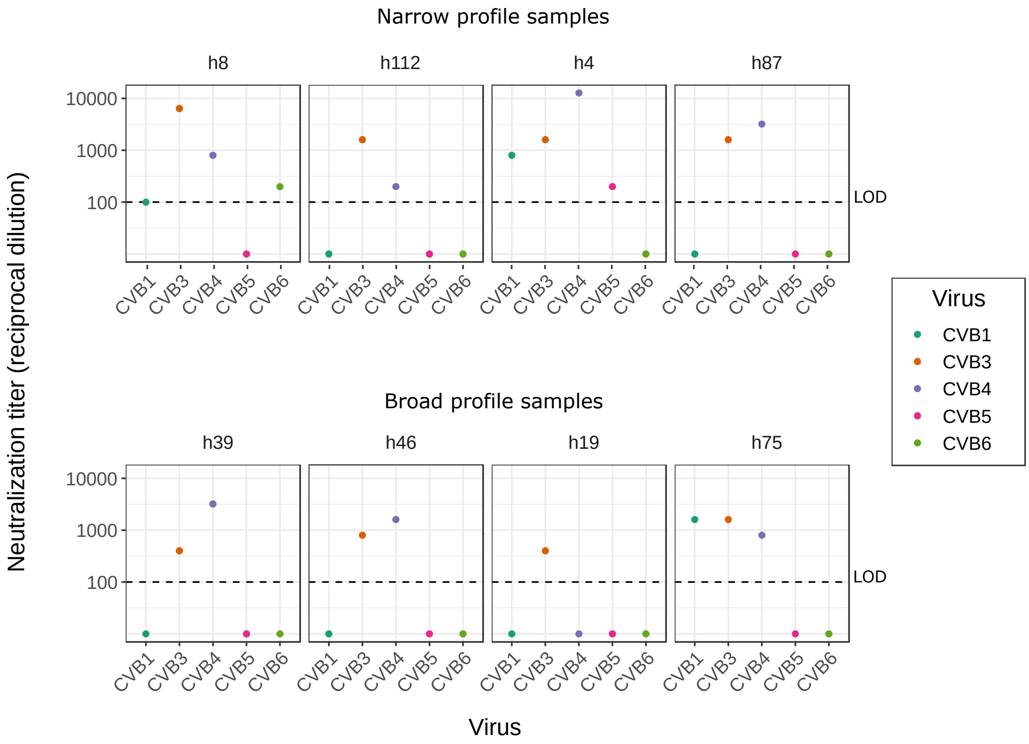

Figure S7. Neutralization titers of the top 8 human sera samples for different coxsackievirus B serotypes. Neutralization titer was determined as the reciprocal of the highest dilution at which at least 50% of wells showed complete inhibition of CPE. Of note, the CVB3 strain used in this assay is a clinical isolate and not the laboratory strain used in the remainder of the manuscript. Data available on GitHub (https://github.com/RGellerLab/CVB3-Antigenic-Profiling/blob/main/8_neutralization_results/CVBs_neutralization_results.csv)

Supplementary tables

Table S1. Related to Figures 1-4. Surviving fraction of viral populations after neutralization with the mAb or the mouse and human polyclonal sera. Antibody concentration used and the number of replicates (rep) are indicated.

Library	Ab	rep	Ab concentration (µL diluted in 100µL)	Surviving fraction
L1	h4	1	1.25	2.50
L1	h4	2	1.25	2.97
L1	h8	1	1.25	0.73
L1	h8	2	1.25	0.91
L1	h39	1	5	1.70
L1	h39	2	5	3.40
L1	h46	1	5	0.87
L1	h46	2	5	1.11
L1	m1	1	5	4.09
L1	m1	2	5	11.69
L1	m2	1	5	43.74
L1	m2	2	5	12.18
L1	m3	1	5	10.53
L1	m3	2	5	12.45
L1	mAb	1	7.5	3.91
L1	mAb	2	7.5	1.62
L1	h112	1	5	0.92
L1	h19	1	5	0.44
L1	h75	1	5	0.59
L2	h4	1	1.25	1.81
L2	h4	2	1.25	2.69
L2	h8	1	1.25	2.84
L2	h8	2	1.25	1.22
L2	h39	1	5	4.12
L2	h39	2	5	4.77
L2	h46	1	5	2.43
L2	h46	2	5	1.75
L2	m1	1	5	3.31
L2	m1	2	5	2.39
L2	m2	1	5	8.67
L2	m2	2	5	12.01
L2	m3	1	5	5.56
L2	m3	2	5	5.69
L2	mAb	1	7.5	2.32
L2	mAb	2	7.5	1.75
L2	h87	1	1.5	0.31
L2	h112	1	5	2.37
L2	h19	1	5	1.14
L2	h75	1	5	2.75

Table S2. Related to Figures 1 and 2. Main sites of escape of the mAb and mice antigenic profiles. Surface exposed residues with a positive differential selection value above the mean+2SD per sample (SS: secondary structure element).

Sample(s)	Site	VP	Position	SS
m3	211	2	142	VP2 EF loop
m2	212	2	143	VP2 EF helix
m3	225	2	156	VP2 EF loop
mAb, m3	227	2	158	VP2 EF loop
mAb	228	2	159	VP2 EF loop
mAb	229	2	160	VP2 EF loop
mAb	230	2	161	VP2 EF loop
m3	232	2	163	VP2 EF loop
m3	236	2	167	VP2 EF loop
mAb	242	2	173	VP2 EF helix
m3	299	2	230	VP2 H
m3	390	3	58	VP3 AB loop
mAb	391	3	59	VP3 AB helix
mAb	392	3	60	VP3 AB helix
mAb, m3	393	3	61	VP3 AB helix
mAb	396	3	64	VP3 AB loop
m3	401	3	69	VP3 AB loop
m2, m3	513	3	181	VP3 G2H loop
m3	541	3	209	VP3 I
m1, m2, m3	650	1	80	VP1 B
m1, m2, m3	652	1	82	VP1 BC loop
m1, m2, m3	654	1	84	VP1 BC loop
m1, m2, m3	655	1	85	VP1 BC loop
m1	656	1	86	VP1 BC loop
m2	698	1	128	VP1 DE loop
m3	699	1	129	VP1 DE loop
m2	701	1	131	VP1 DE loop
m2	702	1	132	VP1 DE loop
m1, m2, m3	723	1	153	VP1 EF loop
m3	777	1	207	VP1 G2H helix
m3	797	1	227	VP1 HI loop
m1, m2, m3	800	1	230	VP1 I
m2	830	1	260	VP1 C-term loop
m1, m3	834	1	264	VP1 C-term loop
m2, m3	840	1	270	VP1 C-term loop
m3	843	1	273	VP1 C-term loop
m3	846	1	276	VP1 C-term loop
m2, m3	847	1	277	VP1 C-term loop
m2	849	1	279	VP1 C-term loop

Table S3. Related to Figures 3 and 4. Main sites of escape of the human antigenic profiles. Surface exposed residues with a positive differential selection value above the mean+2SD per sample (SS: secondary structure element).

Samples	Site	VP	Position	SS
h39, h75	143	2	74	VP2 BC loop
h46	208	2	139	VP2 EF loop
h19	212	2	143	VP2 EF helix
h75, h87	213	2	144	VP2 EF helix
h8, h46, h75	221	2	152	VP2 EF loop
h87	222	2	153	VP2 EF loop
h8, h19	225	2	156	VP2 EF loop
h8, h19	227	2	158	VP2 EF loop
h8, h87	230	2	161	VP2 EF loop
h19	231	2	162	VP2 EF loop
h8	239	2	170	VP2 EF loop
h19	242	2	173	VP2 EF helix
h4, h39, h46	309	2	240	VP2 HI loop
h19	331	2	262	VP2 C-term loop
h19	388	3	56	VP3 AB loop
h39	390	3	58	VP3 AB loop
h19	401	3	69	VP3 AB loop
h19, h75	410	3	78	VP3 BC loop
h87	418	3	86	VP3 C
h4	423	3	91	VP3 CD loop
h112	473	3	141	VP3 EF loop
h75, h112	475	3	143	VP3 EF loop
h112	476	3	144	VP3 EF loop
h4, h19, h75	512	3	180	VP3 G2H loop
h87	513	3	181	VP3 G2H loop
h87	515	3	183	VP3 G2H loop
h87	524	3	192	VP3 H
h46	535	3	203	VP3 H loop
h87	536	3	204	VP3 H loop
h46	561	3	229	VP3 C-term loop
h4	564	3	232	VP3 C-term loop
h39	647	1	77	VP1 B
h39	648	1	78	VP1 B
h39, h112	650	1	80	VP1 B
h39	652	1	82	VP1 BC loop
h39	656	1	86	VP1 BC loop
h4, h75	661	1	91	VP1 C
h4	666	1	96	VP1 CD loop
h4	693	1	123	VP1 D
h4, h39, h46, h75	694	1	124	VP1 D
h39	695	1	125	VP1 DE loop
h4, h39, h75	696	1	126	VP1 DE loop
h75	698	1	128	VP1 DE loop
h4, h46	699	1	129	VP1 DE loop
h39, h46	700	1	130	VP1 DE loop
h4, h39, h46	701	1	131	VP1 DE loop
h4, h39, h46, h75	702	1	132	VP1 DE loop
h4, h39, h46	703	1	133	VP1 DE loop
h4, h46	704	1	134	VP1 DE loop
h4, h39	705	1	135	VP1 DE loop
h4, h19	706	1	136	VP1 DE loop
h39	722	1	152	VP1 EF loop
h4, h39, h19, h75	723	1	153	VP1 EF loop
h4, h46	724	1	154	VP1 EF loop
h4	740	1	170	VP1 F
h46	742	1	172	VP1 FG1 loop
h19	772	1	202	VP1 G2H loop
h39	774	1	204	VP1 G2H loop
h4	779	1	209	VP1 G2H helix
h4	782	1	212	VP1 G2H loop
h39	790	1	220	VP1 H
h39	793	1	223	VP1 HI loop
h4	796	1	226	VP1 HI loop
h4, h39, h46	797	1	227	VP1 HI loop
h4, h39, h46	798	1	228	VP1 HI loop
h75, h112	800	1	230	VP1 I
h4	802	1	232	VP1 I
h4, h75	827	1	257	VP1 C-term loop
h4, h46, h75	829	1	259	VP1 C-term loop
h46	831	1	261	VP1 C-term loop
h39, h75	840	1	270	VP1 C-term loop
h75, h87	843	1	273	VP1 C-term loop
h19	846	1	276	VP1 C-term loop
h87, h112	847	1	277	VP1 C-term loop
h112	848	1	278	VP1 C-term loop
h19	849	1	279	VP1 C-term loop

Table S4. Related to Figure 5. Comparison between different machine learning algorithms.

			Prior Prob. + Prob. Recal	Prior Prob. + No Prob. Recal	No Prior Prob. + Prob. Recal	No Prior Prob. + No Prob. Recal
Machine Learning Algorithm	Neural Network	Accuracy	$95.2 \pm 0.6\%$	$95.1 \pm 0.6\%$	$91.8 \pm 0.7\%$	$92.9 \pm 0.7\%$
		F_1-score	**no escape:** $97.5 \pm 0.3\%$ **escape**: $17 \pm 5\%$	**no escape:** $97.5 \pm 0.3\%$ **escape**: $12 \pm 4\%$	**no escape**: $95.7 \pm 0.5\%$ **escape**: $18 \pm 3\%$	**no escape**: $96.3 \pm 0.4\%$ **escape**: $22 \pm 4\%$
		Precision	**no escape**: $96.7 \pm 0.5\%$ **escape**: $24 \pm 8\%$	**no escape**: $96.5 \pm 0.5\%$ **escape**: $19 \pm 8\%$	**no escape**: $97.0 \pm 0.5\%$ **escape**: $14 \pm 4\%$	**no escape**: $97.1 \pm 0.5\%$ **escape**: $18 \pm 4\%$
		ROC AUC	**no escape**: $81 \pm 1\%$ **escape**: $81 \pm 1\%$	**no escape**: $83 \pm 1\%$ **escape**: $83 \pm 1\%$	**no escape**: $82 \pm 1\%$ **escape**: $82 \pm 1\%$	**no escape**: $81 \pm 1\%$ **escape**: $81 \pm 1\%$
	Support Vector Machine	Accuracy	$95.4 \pm 0.5\%$	$95.2 \pm 0.6\%$	$90.1 \pm 0.8\%$	$92.3 \pm 0.7\%$
		F_1-score	**no escape**: $97.6 \pm 0.3\%$ **escape**: $28 \pm 5\%$	**no escape**: $97.5 \pm 0.3\%$ **escape**: $29 \pm 5\%$	**no escape**: $95.0 \pm 0.4\%$ **escape**: $26 \pm 3\%$	**no escape**: $95.9 \pm 0.4\%$ **escape**: $29 \pm 4\%$
		Precision	**no escape**: $97.1 \pm 0.4\%$ **escape**: $34 \pm 8\%$	**no escape**: $97.1 \pm 0.4\%$ **escape**: $32 \pm 7\%$	**no escape**: $97.7 \pm 0.4\%$ **escape**: $19 \pm 3\%$	**no escape**: $97.7 \pm 0.4\%$ **escape**: $22 \pm 4\%$
		ROC AUC	**no escape**: $84.8 \pm 0.9\%$ **escape**: $84.8 \pm 0.9\%$	**no escape**: $85.1 \pm 0.9\%$ **escape**: $85.1 \pm 0.9\%$	**no escape**: $85.5 \pm 0.9\%$ **escape**: $85.5 \pm 0.9\%$	**no escape**: $84.8 \pm 0.9\%$ **escape**: $84.8 \pm 0.9\%$
	Random Forest	Accuracy	$95.7 \pm 0.5\%$	$96.3 \pm 0.5\%$	$92.8 \pm 0.7\%$	$93.2 \pm 0.7\%$
		F_1-score	**no escape**: $97.8 \pm 0.3\%$ **escape**: $26 \pm 5\%$	**no escape**: $98.1 \pm 0.3\%$ **escape**: 0%	**no escape**: $96.2 \pm 0.4\%$ **escape**: $36 \pm 4\%$	**no escape**: $96.4 \pm 0.4\%$ **escape**: $36 \pm 4\%$
		Precision	**no escape**: $97.0 \pm 0.5\%$ **escape**: $37 \pm 9\%$	**no escape**: $96.3 \pm 0.5\%$ **escape**: 0%	**no escape**: $98.1 \pm 0.4\%$ **escape**: $27 \pm 4\%$	**no escape**: $98.0 \pm 0.4\%$ **escape**: $28 \pm 5\%$
		ROC AUC	**no escape**: $90.7 \pm 0.8\%$ **escape**: $90.6 \pm 0.8\%$	**no escape**: $90.1 \pm 0.8\%$ **escape**: $89.9 \pm 0.8\%$	**no escape**: $90.7 \pm 0.8\%$ **escape**: $90.6 \pm 0.8\%$	**no escape**: $90.1 \pm 0.8\%$ **escape**: $89.9 \pm 0.8\%$

Table S5. Related to Figure 5. Classification metrics of the Random Forest classifiers tested.

	All features + no reclassification		All features + reclassification (27 cases)		Top 7 features + reclassification	
	**Training**	**Testing**	**Training**	**Testing**	**Training**	**Testing**
**Accuracy**	$99.72 \pm 0.06\%$	$95.7 \pm 0.5\%$	$99.84 \pm 0.04\%$	$96.1 \pm 0.5\%$	$99.72 \pm 0.06\%$	$96.8 \pm 0.5\%$
**F_1-score**	**no escape:** $99.72 \pm 0.06\%$ **escape:** $99.72 \pm 0.06\%$	**no escape:** $97.8 \pm 0.3\%$ **escape:** $26 \pm 5\%$	**no escape:** $99.84 \pm 0.04\%$ **escape:** $99.84 \pm 0.04\%$	**no escape:** $98.0 \pm 0.3\%$ **escape:** $50 \pm 5\%$	**no escape:** $99.72 \pm 0.06\%$ **escape:** $99.72 \pm 0.06\%$	**no escape:** $98.4 \pm 0.2\%$ **escape:** $50 \pm 5\%$
**Precision**	**no escape:** 100% **escape:** $99.5 \pm 0.1\%$	**no escape:** $97.07 \pm 0.4\%$ **escape:** $37 \pm 9\%$	**no escape:** 100% **escape:** $99.69 \pm 0.09\%$	**no escape:** $97.7 \pm 0.4\%$ **escape:** $54 \pm 7\%$	**no escape:** 100% **escape:** $99.5 \pm 0.01\%$	**no escape:** $97.4 \pm 0.4\%$ **escape:** $72 \pm 8\%$
**ROC AUC**	**no escape:** $99.99 \pm 0.01\%$ **escape:** $99.99 \pm 0.01\%$	**no escape:** $90.7 \pm 0.8\%$ **escape:** $90.6 \pm 0.8\%$	**no escape:** $99.999 \pm 0.002\%$ **escape:** $99.999 \pm 0.002\%$	**no escape:** $93.4 \pm 0.7\%$ **escape:** $93.3 \pm 0.7\%$	**no escape:** $99.993 \pm 0.009\%$ **escape:** $99.99 \pm 0.01\%$	**no escape:** $93.2 \pm 0.7\%$ **escape:** $93.1 \pm 0.7\%$

Table S6. Related to figure 6 and S6. Secondary structure elements of the picornavirus capsid involved in antigenic sites of different enterovirus serotypes. Data for poliovirus and human rhinovirus was obtained from the review of Mateu, M.G. (Virus Res., 1995) and derives from different studies that determined the antigenic sites of these viruses by selection of escape mutants using monoclonal antibodies. Data for CVB3 was obtained from the antigenic profiling experiments performed in this work and includes secondary structure elements containing ≥ 3 escape sites (highlighted in bold in Figure S6). Structural elements present as antigenic sites in ≥ 3 serotypes are highlighted in bold. [a] Antigenic sites were named according to standard nomenclature, as described in Mateu, M.G. (Virus Res., 1995). The nomenclature of CVB3 antigenic sites was based on homology to PV1 antigenic regions.

**Capsid**		**Antigenic sites[a]**					
**Axis**	**Secondary structure element**	**Poliovirus**			**Rhinovirus**		**Coxsackievirus**
		**1**	**2**	**3**	**14**	**2**	**B3**
**5X**	**VP1 B-C**	1	A	1	Ia, Ib	A	1
	VP1 H-I				Ia		1
	**VP1 D-E**	1		1	Ib		1
	VP1 E-F		A	1			1
**2X**	**VP1 G-H**	2	B				2
	**VP2 E-F**	2	C, D	2	II	B	2
	VP2 C_T	2					
**3X**	VP2 B-C	3	D				
	VP2 H-I	3	D			C	
	**VP3 A-B**	3	D	3		B	3
	VP3 B-C	3		4	III		
	VP3 H-I	3	D		III		
	**VP1 C_T**			3	III	B	3
	VP3 E-F	3					3
	VP3 G-H						3
