# Supplementary material for: Comprehensive profiling of neutralizing polyclonal sera targeting coxsackievirus B3
Source: Nat Commun. 2023 Oct 12;14:6417. doi: 10.1038/s41467-023-42144-2 (PMC10570382; doi:10.1038/s41467-023-42144-2)
Supplement: Supplementary file 3 — Reporting Summary [file 41467_2023_42144_MOESM3_ESM.pdf]

nature portfolio

Corresponding author(s): Beatriz Alvarez-Rodriguez, Javier Buceta, Ron Geller

Last updated by author(s): Sep 25, 2023

Reporting Summary

Nature Portfolio wishes to improve the reproducibility of the work that we publish. This form provides structure for consistency and transparency in reporting. For further information on Nature Portfolio policies, see our Editorial Policies and the Editorial Policy Checklist.

Statistics

For all statistical analyses, confirm that the following items are present in the figure legend, table legend, main text, or Methods section.

n/a	Confirmed	
☐	☒	The exact sample size (*n*) for each experimental group/condition, given as a discrete number and unit of measurement
☐	☒	A statement on whether measurements were taken from distinct samples or whether the same sample was measured repeatedly
☐	☒	The statistical test(s) used AND whether they are one- or two-sided<br>*Only common tests should be described solely by name; describe more complex techniques in the Methods section.*
☒	☐	A description of all covariates tested
☐	☒	A description of any assumptions or corrections, such as tests of normality and adjustment for multiple comparisons
☐	☒	A full description of the statistical parameters including central tendency (e.g. means) or other basic estimates (e.g. regression coefficient) AND variation (e.g. standard deviation) or associated estimates of uncertainty (e.g. confidence intervals)
☐	☒	For null hypothesis testing, the test statistic (e.g. *F*, *t*, *r*) with confidence intervals, effect sizes, degrees of freedom and *P* value noted<br>*Give P values as exact values whenever suitable.*
☒	☐	For Bayesian analysis, information on the choice of priors and Markov chain Monte Carlo settings
☒	☐	For hierarchical and complex designs, identification of the appropriate level for tests and full reporting of outcomes
☐	☒	Estimates of effect sizes (e.g. Cohen's *d*, Pearson's *r*), indicating how they were calculated

Our web collection on statistics for biologists contains articles on many of the points above.

Software and code

Policy information about availability of computer code

Data collection	All this information is present in the Bioinformatic analyses methods section and/or other methods sections.
Data analysis	All information and primary data is available either in the manuscript (methods section) or our Github, as indicated in the manuscript.

For manuscripts utilizing custom algorithms or software that are central to the research but not yet described in published literature, software must be made available to editors and reviewers. We strongly encourage code deposition in a community repository (e.g. GitHub). See the Nature Portfolio guidelines for submitting code & software for further information.

Data

Policy information about availability of data

All manuscripts must include a data availability statement. This statement should provide the following information, where applicable:

- Accession codes, unique identifiers, or web links for publicly available datasets
- A description of any restrictions on data availability
- For clinical datasets or third party data, please ensure that the statement adheres to our policy

Sequencing data have been uploaded to SRA (BioProject PRJNA779606, SRA: SRP345591). The following supplementary files are available on GitHub (https://github.com/RGellerLab/CVB3-Antigenic-Profiling or at this DOI https://doi.org/10.5281/zenodo.8278367

Human research participants

Policy information about studies involving human research participants and Sex and Gender in Research.

Reporting on sex and gender	Sera from both sexes were evaluated for neutralization activity at nearly equal proporations.
Population characteristics	Adult population of equal gender proportions. Anonymous data on date of collection, age, and sex of donors is available as a supplementary table.
Recruitment	Samples are from an existing collection of a biobank.
Ethics oversight	Ethics committee of IBSP-CV Biobank

Note that full information on the approval of the study protocol must also be provided in the manuscript.

Field-specific reporting

Please select the one below that is the best fit for your research. If you are not sure, read the appropriate sections before making your selection.

[x] Life sciences   [ ] Behavioural & social sciences   [ ] Ecological, evolutionary & environmental sciences

For a reference copy of the document with all sections, see nature.com/documents/nr-reporting-summary-flat.pdf

Life sciences study design

All studies must disclose on these points even when the disclosure is negative.

Sample size	We aimed to identify 8 highly neutralizing sera to do the antigenic profiling on, which we considered sufficient to get a general idea of how polyclonal sera target the virus. For this, we first screened 60 sera and subsequently requested an additional 80 sera to reach 8 highly neutralizing sera.
Data exclusions	None
Replication	The number of replicates are indicated in the text. Neutralization data was performed in duplicates. Profiling data was performed either as duplicates with two different populations or once with two different populations as indicated. Data represent the average, as indicated.
Randomization	We don't have distinct groups.
Blinding	All animals were treated equally and hence there was no need for blinding.

Reporting for specific materials, systems and methods

We require information from authors about some types of materials, experimental systems and methods used in many studies. Here, indicate whether each material, system or method listed is relevant to your study. If you are not sure if a list item applies to your research, read the appropriate section before selecting a response.

Materials & experimental systems

n/a	Involved in the study	
[ ]	[x]	Antibodies
[ ]	[x]	Eukaryotic cell lines
[x]	[ ]	Palaeontology and archaeology
[ ]	[x]	Animals and other organisms
[x]	[ ]	Clinical data
[x]	[ ]	Dual use research of concern

Methods

n/a	Involved in the study	
[x]	[ ]	ChIP-seq
[x]	[ ]	Flow cytometry
[x]	[ ]	MRI-based neuroimaging

Antibodies

Antibodies used	The anti-Coxsackievirus B3 monoclonal antibody was obtained from Merck (clone 280-5F-4E-5E, MAB948).
Validation	We internally validate the antibody in our paper and see agreement with published results, as cited in the paper.

Eukaryotic cell lines

Policy information about cell lines and Sex and Gender in Research

Cell line source(s)	ATCC for Hela-H1 (CRL-1958) and HEK293 (CRL-3216)
Authentication	None
Mycoplasma contamination	Tested periodically to be free of mycoplasma
Commonly misidentified lines (See ICLAC register)	None

Animals and other research organisms

Policy information about studies involving animals; ARRIVE guidelines recommended for reporting animal research, and Sex and Gender in Research

Laboratory animals	5-week old Balb/C male mice. Acquired commercially.
Wild animals	None
Reporting on sex	Testing is only in males due to differences in infection outcomes between the sexes.
Field-collected samples	None
Ethics oversight	Animal work was approved by the Valencian government's ethics committee (approval 2019/VSC/PEA/0151). Experiments with infectious viruses and genetically modified organisms were approved by the Spanish committee on genetically modified organisms, the University of Valencia's biosafety committee, and the institutes biosafety committee.

Note that full information on the approval of the study protocol must also be provided in the manuscript.
