# Supplementary material for: Comprehensive profiling of neutralizing polyclonal sera targeting coxsackievirus B3
Source: Nat Commun. 2023 Oct 12;14:6417. doi: 10.1038/s41467-023-42144-2 (PMC10570382; doi:10.1038/s41467-023-42144-2)
Supplement: Supplementary file 4 — Description of Additional Supplementary Files [file 41467_2023_42144_MOESM4_ESM.pdf]

Description of additional supplementary files

Supplementary Data 1 : Alignment of the capsid region of all full-length CVB3 genomic sequences available on bv-brc.org obtained from human sources, downloaded on July 25, 2022.
